# Incidence Rates of Root Rot in Sweetpotato Caused by Cultivation Soil and Soil Microorganisms During Storage Periods

**DOI:** 10.3389/fpls.2022.897590

**Published:** 2022-05-03

**Authors:** Sujung Kim, Tae Hwa Kim, Mi-Nam Chung, YeongHoon Lee, Im Been Lee, HyeongUn Lee, Won Park

**Affiliations:** Bioenergy Crop Research Institute, National Institute of Crop Science, Rural Development Administration, Muan, South Korea

**Keywords:** sweetpotato, root rot, microbiome, Fusarium, storage

## Abstract

Sweetpotatoes require a storage period for year-round use and improved sweetness by starch degradation. However, long-term storage can cause root rot, and a large amount of sweetpotatoes can be discarded. Root rot is typically caused by pathogenic soil-borne *Fusarium* spp., and the development of root rot induced by the characteristics of cultivating soil in stored sweetpotato has not yet been identified. In this study, the effect of *Fusarium* spp. and microbial community in the cultivated soil on the root rot of sweetpotatoes was to be elucidated. Wounded sweetpotato were treated in soil cultures inoculated with *F. solani* or *F. oxysporum* for 2 days, and showed symptoms of root rot after 2 months of storage. The three study fields (Naju, Yeongam A, and B) were subjected to the same curing and storage treatments after harvest, and the incidence of root rot was 1.7- to 1.8-fold different after 3 months of storage. Across the three fields, concentrations of *Fusarium* spp. and of microbial communities differed according to the cultivation soil and period. In particular, Naju, which had the lowest incidence of root rot, had the lowest concentration of *Fusarium* spp. before harvest, and the smallest change in diversity of the microbial community during the cultivation period. However, tuberous roots harvested from the fields showed no significant differences in antioxidant activity or lesion size with the treatment of 10^6^ conidia/ml *F. solani.* By solidifying the importance of cultivating soil and related microorganisms in the advancement of root rot of sweetpotato, our results may aid in preventing the decrease in the yield of cultivated sweetpotatoes through root rot control.

## Introduction

The marketability of sweetpotato relies on their sweetness, which is enhanced through the increase of free sugar content obtained during storage (Adu-Kwarteng et al., 2014). Therefore, sweetpotatoes must be stored for long periods after harvesting for their year-round use and to allow for the increase of free sugar content *via* starch degradation during these periods.

Root rot forms dry brown lesions on the epidermis and cortex of plants and has been found to be induced by pathogenic soil-borne *Fusarium* spp. in many crops (Ozbay and Newman, 2004; Beccari et al., 2011; Chang et al., 2015). In sweetpotatoes, root rot is induced by *F. oxysporum* and *F. solani* and is known to occur during long storage periods (Scruggs and Quesada-Ocampo, 2016; Yang et al., 2018). Because diseases caused during storage reduce sweetpotato yield, their control is very important. Root rot of sweetpotato is induced by soil pathogens that invade through wounds after harvest (Ray and Ravi, 2005). However, there are few studies on the induction of root rot by *Fusarium* spp. in the soil through wounds of sweetpotato.

Soil microorganisms have many effects on plants. Soils with abundant beneficial microorganisms enhance plant growth and that with abundant harmful microorganisms inhibit plant growth (Misk and Franco, 2011; Palaniyandi et al., 2013). Sweetpotatoes are also known to interact with soil microbes. The genotype of sweetpotato affects soil bacteria involved in phosphate mineralization and nitrogen fixation (Marques et al., 2019). Phosphate fertilizers affect the bacterial community in sweetpotato cultivation (Minemba et al., 2020). In addition, continuous cropping of sweetpotatoes increases soil-borne diseases, such as root rot, and decreases sweetpotato yield by reducing the amount of beneficial fungi and increasing harmful ones (Gao et al., 2019).

Plants have some mechanisms of defense response to abiotic stress or biotic stress, and mechanisms for antioxidant enzymes and antioxidant activity have been shown in sweetpotatoes as well against abiotic stress, such as drought stress or salt stress (Lin et al., 2006; Kim et al., 2009, 2013). In addition, sweetpotatoes have an antioxidant defense response to black rot caused by *Ceratocystis fimbriata* (Mohsin et al., 2021). However, the defense response of sweetpotatoes to root rot caused by *Fusarium* spp. is relatively unknown.

In this study, we aimed to observe symptoms of root rot in stored sweetpotatoes induced by *Fusarium* spp. in a cultured soil treatment for 2 months. In addition, differences in the incidence rates of root rot and the change in the microbial community according to the cultivating soil were shown. We showed that antioxidant activity and degree of symptoms induced by *Fusarium* spp. were not significantly different in sweetpotatoes harvested from the three investigated cultivation fields. Therefore, results of this study suggest the importance of soil cultivation and soil microbial management to promote sweetpotato yield through root rot control.

## Materials and Methods

### Soil Inoculation and Sweetpotato Infection

Pathogen-cultured soil was used to confirm that the root rot of sweetpotatoes was induced during the storage period. Soil inoculation with *Fusarium* spp. was performed by modifying the method of Arias et al. (2013), who studied root rot in soybeans (Supplementary Figure 1). The soil consisted of 380 ml cornmeal, 1900 ml sand, and 110 ml sterile distilled water (SDW), and was sterilized twice at 121°C for 45 min. After cooling the sterile soil, a suspension of 2 ml of *F. solani* (NCBI accession number MZ930186) or *F. oxysporum* isolate SPL18019 (Paul et al., 2020) with 10^6^ conidia/ml was added (for the soil culture efficiency test, concentrations of 10^4,^ 10^5,^ 10^6^ conidia/ml). The mixed soil was then cultured for 6 days (26°C, dark conditions), after which the *Fusarium*-cultured soil was further mixed with 4,560 ml and 2,280 ml sterile sand and soil, respectively, at 121°C for 45 min (twice), in a 3:1 ratio (v/v). As a control, sterile soil containing SDW was used instead of the *Fusarium* isolate suspension with 10^6^ conidia/ml. Sweetpotatoes were sterilized with NaClO 1%, ethanol, and SDW before use. Wounded sweetpotato tubers were treated in the final mixed soil for 2 days (26°C). Subsequently, the tubers were kept in storage (13°C, 85–90%) for 2 months.

### Soil Sampling and Disease Incidence

For this study, we selected three sweetpotato cultivation fields (Naju, Yeongam A, and Yeongam B) within a radius of 14 km that was managed by one farmer, to ensure that consistent conditions for curing and storage after harvest. However, soil conditions for growing were different. The soil in the cultivation area was stored at 4°C for *Fusarium* spp. concentration and microbiome analysis, which were performed within a week.

The incidence rate of root rot during storage in sweetpotatoes harvested from the three regions was investigated. Sweetpotatoes stored for 3 months at 13°C (85–90%) were used to investigate the disease incidence rate in three replicates of five biological replicates. Moreover, artificial infection was performed on sweetpotatoes from each field, and the lesion diameter was measured to compare differences in resistance of sweetpotatoes from each field. The epidermis of the tubers was wounded and inoculated with 10 μl of *F. solani* (NCBI accession number MZ930186) with 10^6^ conidia/ml, and the diameter of the black decay was measured two weeks later.

### *Fusarium* spp. Concentration in Soil

The *Fusarium* concentrations of pathogen-infected soil and sweetpotato-cultivating soil were measured using Komada’s selective medium (MB cell, Seoul, Korea; Komada, 1975). Komada agar was prepared according to the manufacturer’s instructions. The soil and SDW were mixed and diluted to the concentrations of 100, 10, 1, and 0.1% (w/v). A 100 μl of suspension was spread on Komada agar and incubated for 10 days (25°C). For artificially infected soil, the number of colonies on Komada agar was measured. In contrast, in the soil collected from the three study fields, the number of *Fusarium* species was confirmed through BLAST analysis of the internal transcribed spacer (ITS) sequence of each colony. Total genomic DNA of isolated colonies was extracted using a Solg Genomic DNA Prep Kit for fungi (Solgent Co., Ltd., Korea). PCR amplification of genomic DNA was performed using the GoTaq Flexi DNA polymerase kit (Promega, Wisconsin, United States) based on a modified protocol to a 30 l reaction volume for a final solution and a Bio-Rad T100 thermal cycler (Bio-Rad, United States). The regions were amplified using ITS1F (5′-CTTGGTCATTTAGAGGAAGTAA-3′) and ITS4 (5′-TCCTCCGCTTATTGATATGC-3′). The PCR amplification was performed under the following conditions: initial denaturation at 95°C for 2 min, followed by 35 cycles of denaturation at 95°C for 30 s, annealing at 55°C for 30 s, and a final extension at 72°C for 1 min. The PCR products were sequenced by a commercial sequencing service provider (Macrogen, Korea) in both directions.

### DNA Extraction, Illumina Library Construction, and Sequencing

Microbiome analysis was performed to investigate the change in the comprehensive microbial community according to cultivation time in the three fields with differing incidence rates. In addition, microbiome analysis was performed on both inoculated and uninoculated *F. solani* soils. Microbial communities were analyzed using 16S and 18S rDNA sequences. Microbial DNA was extracted from samples using the SPINeasy DNA kit for soil (MP Biomedicals, Germany) according to the manufacturer’s protocols. 16S and 18S rRNA amplicon libraries were prepared using the LoopSeq^™^ 16S and 18S Long Read Kit (Loop Genomics, California, United States). Each of the 24 genomic DNA samples was fragmented and assigned a barcode adapter according to the 16S and 18S rRNA genes for sequencing on an Illumina platform. The libraries were read on an Illumina NovaSeq6000 (Illumina Inc., San Diego, CA, United States) using a paired-end 2 × 150 bp reading system. Sequencing data were analyzed using the QIIME2 classifier (Caporaso et al., 2010) and Silva database (Quast et al., 2013).

### Antioxidant Activity Based on Root Rot in Sweetpotato

For sample extraction, the total polyphenol content (TPC), 2,2-diphenyl-1-picrylhydrazyl-hydrate (DPPH) scavenging activity, and 2,2′-azino-bis (3-ethylbenzothiazoline-6-sulfonate; ABTS) scavenging activity were investigated following Park et al. (2019). A 25 ml amount of 80% methanol was added to 0.25 g of the dry sample and shaken for 24 h (200 rpm, dark conditions) using Flask shaker (Dasol scientific co., LTD., Korea).

The TPC was determined by modifying the Folin–Ciocalteu method (Singleton et al., 1999). The extraction solution was centrifuged (3,000 rpm, 10 min, room temperature), and 100 μl of the supernatant was mixed with 900 μl of distilled water, 500 μl of 1 N Folin–Ciocalteu reagent (Sigma Co., United States), and 2.5 ml of 20% Na_2_CO_3_ (Daejung, Korea). The mixture was centrifuged (3,000 rpm, 10 min, room temperature) and allowed to react at room temperature for 20 min. Subsequently, the absorbance of the supernatant was measured at 735 nm using a UV–vis spectrophotometer (Biochrom, Libra S22, United Kingdom). Chlorogenic acid (Sigma-Aldrich Co., United States) was used as the standard reagent.

To investigate DPPH scavenging activity, we followed methodology by Chen et al. (2004). The extraction solution was centrifuged (3,000 rpm, 10 min, room temperature) and DPPH (2.5 ml) was added to 1 ml of the supernatant of the extraction solution. The mixture was incubated for 1 min at room temperature, and the absorbance of the mixture was measured at 517 nm using a UV–vis spectrophotometer. The DPPH scavenging activity of the samples was calculated as follows:


$$\mathrm{DPPH scavenging activity}\;\left(\%\right)=\left\{1-\left({\mathrm{Abs}}_{\mathrm{sample}}/{\mathrm{Abs}}_{\mathrm{blank}}\right)\right\}\times 100$$

The ABTS scavenging activity was determined by modifying the method described by Re et al. (1999). The extraction solution was centrifuged (3,000 rpm, 10 min, room temperature) and 50 μl of the supernatant was mixed with 1 ml of the reaction solution. The reaction solution was reacted with 7.4 mM ABTS and 2.6 mM potassium persulfate at 1:1 (v/v) for 24 h and diluted 21-fold so that the absorbance value at 735 nm was 1.5 ± 0.1. The final solution was mixed with the supernatant. The reaction solution was incubated for 30 min at room temperature, and the absorbance of the mixture was measured at 735 nm using a UV–vis spectrophotometer. ABTS scavenging activity of the samples was calculated as follows:


$$\mathrm{ABTS scavenging activity}\;\left(\%\right)=\left\{1-\left(\mathrm{Abs}._{\mathrm{sample}}/\mathrm{Abs}._{\mathrm{blank}}\right)\right\}\times 100$$

Diseased tissues demonstrating symptoms and healthy tissues were divided into infection and control parts, respectively, to measure the antioxidant activity as stress response to *F. solani* in tubers. In addition, tubers showing a gradation between the diseased and healthy tissues was the middle part, and the antioxidant activity of each part was measured.

## Results

### Soil Inoculation of *Fusarium* spp. and Root Rot in Sweetpotato

Although root rot of sweetpotato is known to occur during the storage period, this study identified *Fusarium* spp. as a pathogenic soil-borne fungus causing root rot.

**Figure 1:**
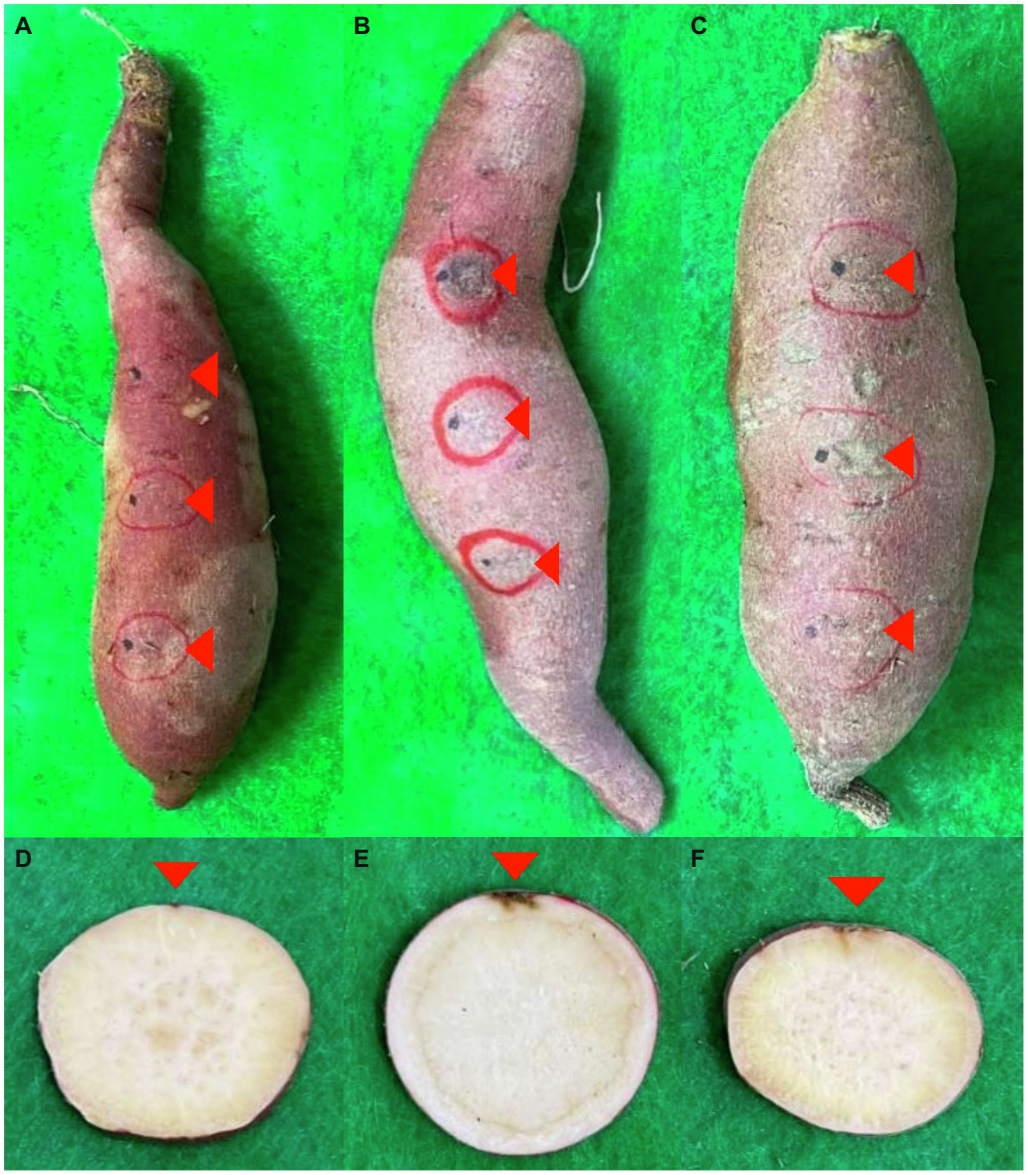
Infection from soil-derived *Fusarium* spp. post-harvest. **(A–F)** indicate symptoms of tubers and cross section, respectively. **(A,D)** are controls. **(B,E)** are *F. solani*. **(C,F)** are *F. oxysporum*. Red triangles represent infected parts.

First, three concentrations (10^4^, 10^5^, and 10^6^ conidia/ml) of *Fusarium*-cultured soil were prepared to confirm the final *F. solani* concentration cultured after 2 days in the soil (Supplementary Figure 2). As the concentration of *F. solani* in the culture soil increased, the number of colonies formed in Komada’s selective medium treated with the culture soil increased after 10 days. In addition, root rot was induced in sweetpotatoes in cultured soil using *F. solani* and *F. oxysporum* (Figure 1). In contrast, the control group had only wounds induced for the invasion of pathogens.

### Selected Fields With Different Incidence Rates of Root Rot in Sweetpotato

**Figure 2:**
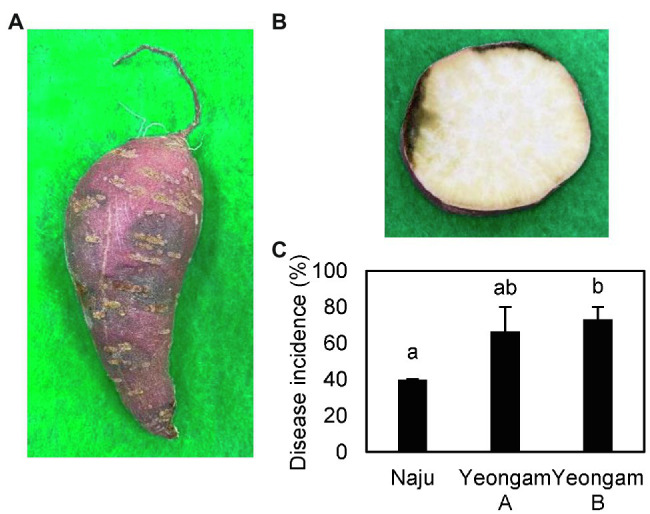
Root rot incidence in the three fields—Naju, Yeongam A, and Yeongam B. **(A)** Lesion of tubers. **(B)** Cross-sectional lesion. **(C)** Severity of root rot of sweetpotatoes in the three regions. Incidence rate was investigated 3 months after storing sweetpotatoes (13°C, 90%). Data are expressed as mean ± S.E. (*n* = 3, five biological replicates). Each value of different letters (a, b) above bars is significantly different by Duncan’s multiple range test at *p* < 0.05.

The conditions of the cultivation areas were different across the three fields in this experiment, but the curing and storage conditions were the same (Table 1). The selected sweetpotato fields have shown different yields because of the different incidence rates of root rot of sweetpotato during storage. The measured incidences of root rot that developed during the storage period of the harvested sweetpotatoes are shown in Figure 2, with the incidence rates differing according to cultivation field. Naju, where a lot of healthy tubers have produced, had an incidence rate of 40%, whereas those of Yeongam A and B were 67 and 73%, respectively.

**Table 1:** Curing and storage information in three fields.

	Location		Curing treatment			Storage treatment	
	Latitude	Longitude	Temperature (°C)	Humidity (%)	Period (days)	Temperature (°C)	Humidity (%)
Naju	34.9495	126.6676	35.5	90	4	13	85
Yeongam A	34.8620	126.6724	35.5	90	4	13	85
Yeongam B	34.8681	126.6627	35.5	90	4	13	85

*Naju and Yeongam A and B had the similar weather conditions because the fields exist within a radius of 14 km. These fields were managed by the one farmer*.

### Fusarium Concentration and Microbial Community in the Three Fields

To measure the concentration of *Fusarium* spp. in the three fields with different incidence rates, the cultivation soil was treated with Komada’s selective medium, and the ITS sequences of the colonies formed in the medium were analyzed (Supplementary Figure 3). *F. oxysporum, F. solani*, and *F. verticillioides*, which have been known to induce root rot in many plants, were found in the three cultivation areas, and the distribution of the fungi differed depending on the cultivation time and place. In Naju, where the incidence of root rot was relatively low, the amount of *Fusarium* spp. before cultivation was higher than that before harvest. In contrast, in Yeongam A and B, which had high incidences of root rot, the amount of *Fusarium* spp. before harvest was higher. However, cultivation time and place were not significantly different.

**Figure 3:**
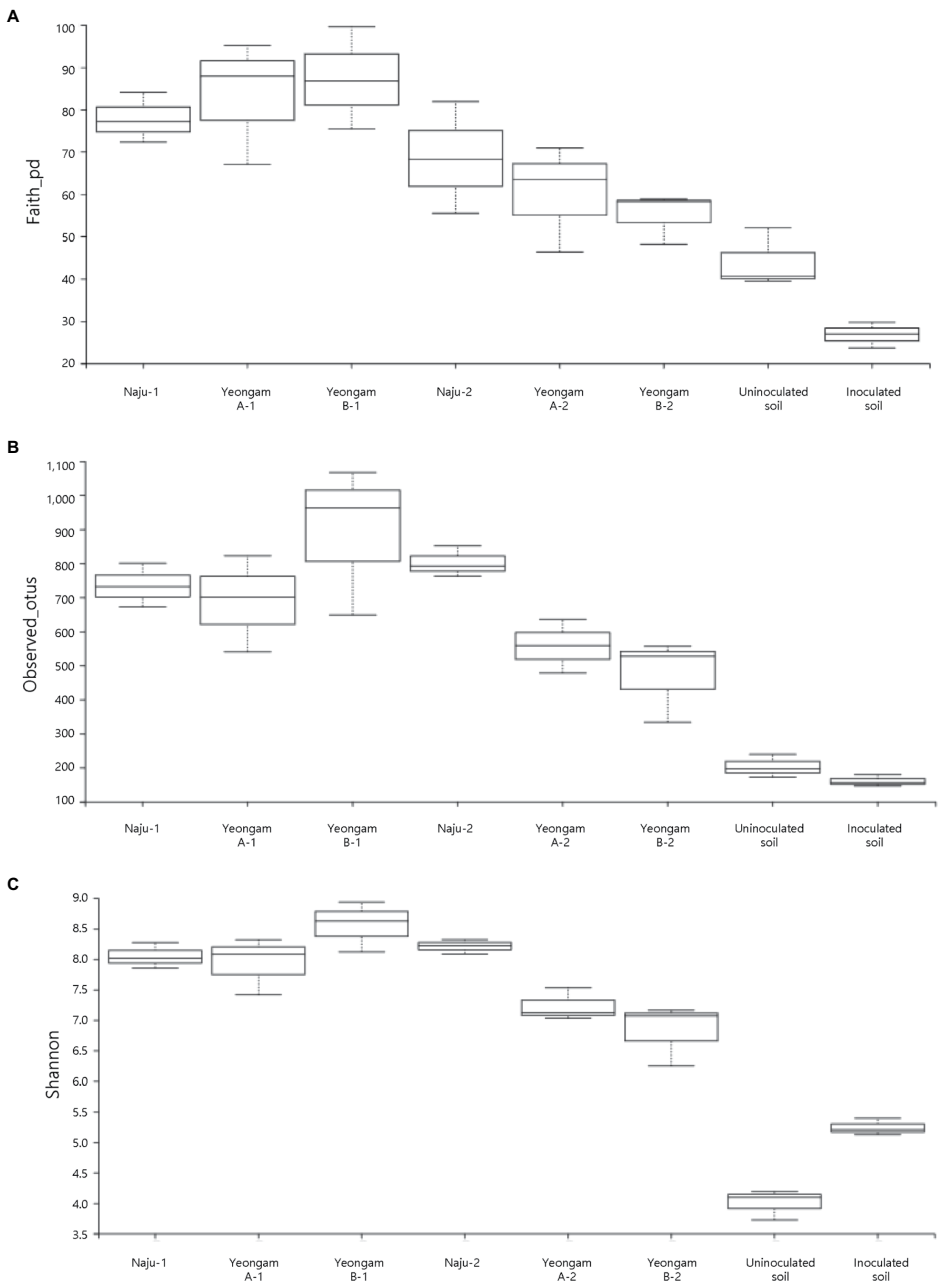
Archaea, bacteria, and eukaryote richness and diversity indices of the three regions and inoculated soil. **(A)** Faith’s PD index. **(B)** Observed OTUs. **(C)** Shannon index. The box graph indicates the interquartile range. The line across the box depicts the median. 1 and 2 indicate before planting and harvest, respectively.

**Figure 4:**
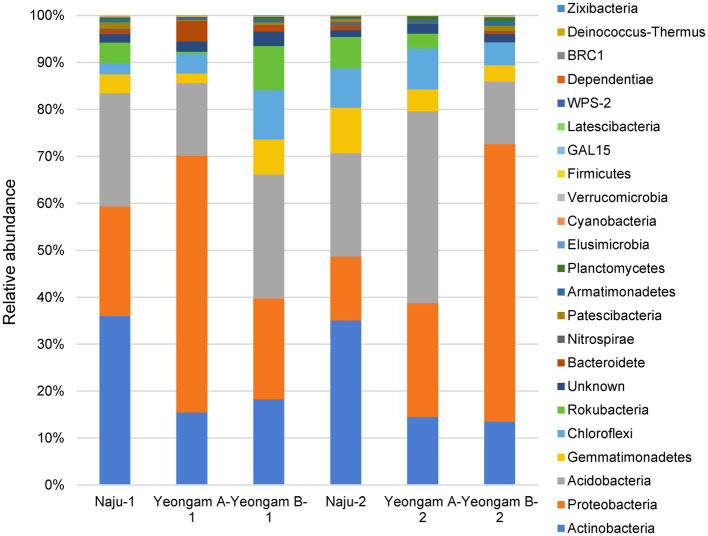
Relative abundance at the phylum level in bacterial communities. Each bar represents the average relative abundance of triplicates.

**Figure 5:**
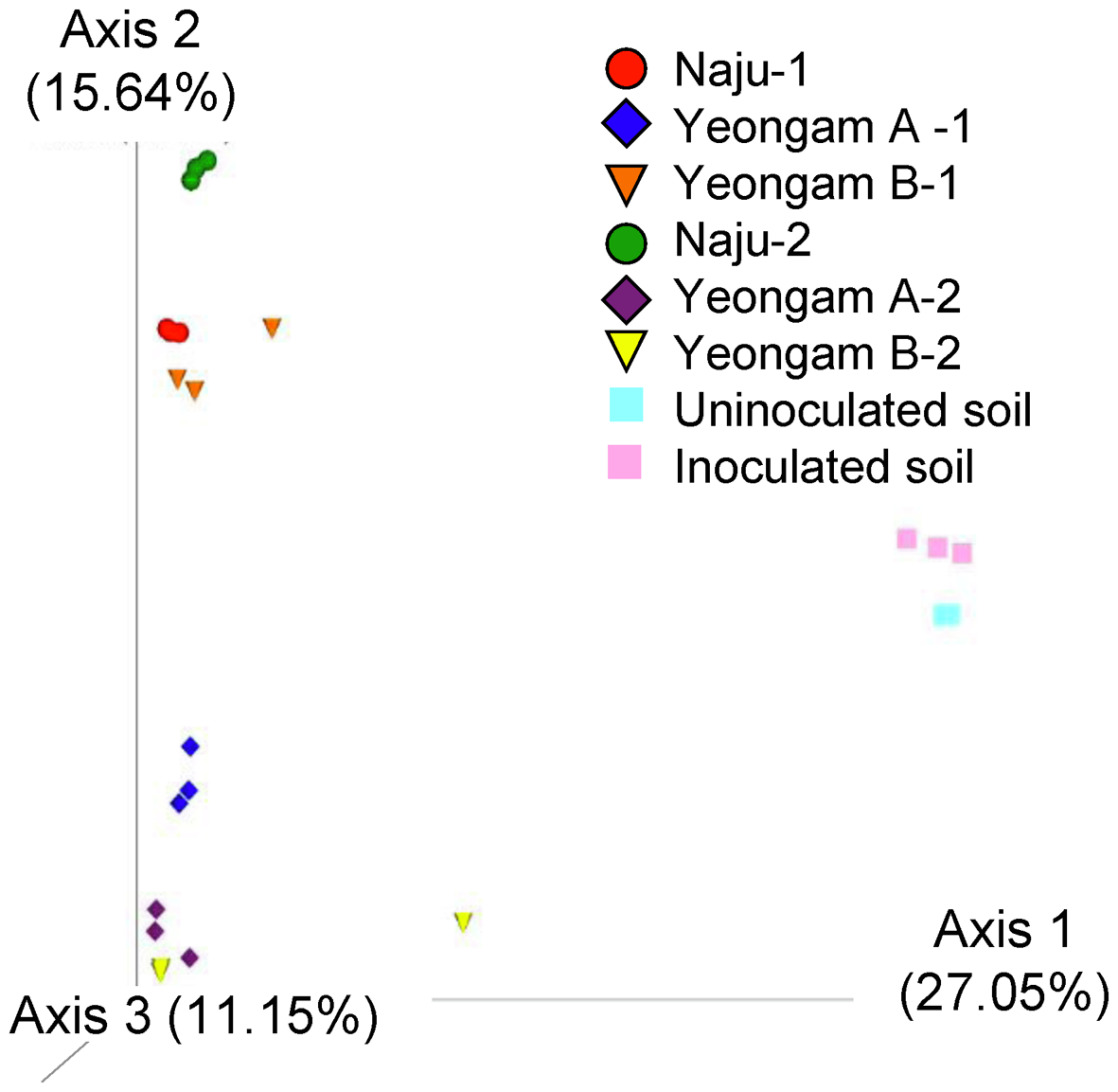
Principal coordinate analysis (PCoA) based on Bray–Curtis dissimilarity of archaea, bacteria, and eukaryote (fungi) from the three fields—Naju, Yeongam A, and Yeongam B, and inoculated soil. Circle, Naju; Diamond, Yeongam A; cone, Yeongam B; Square, uninoculated and inoculated soil. 1 and 2 indicate before planting and harvest, respectively.

Rarefaction curves were prepared based on operational taxonomic units (OTUs) of archaea, bacteria, and eukaryotes including fungi. Rarefaction curves according to the cultivation time of the three fields and cultivating soil of *F. solani* showed saturation with increasing sequence depth, indicating that the sequencing library reflects the sample conditions well (Supplementary Figure 4). To analyze the diversity and richness of microorganisms in the three fields, the number of OTUs, Shannon index, and Faith’s PD index were measured (Figure 3). The number of observed OTUs and Shannon index was higher in Naju before harvest (Naju-2) those in Yeongam A (A-2) and B (B-2). There was no significant change in the number of observed OTUs, Shannon index, and Faith’s PD index before cultivation (Naju-1, Yeongam A-1) and before harvest (Naju-2, Yeongam A-2) in Naju and Yeongam A, but Yeongam B showed a decrease in all three values before harvest (Yeongam B-2) compared to those before cultivation (Yeongam B-1; Kruskal–Wallis test, *p* < 0.05). These results indicate that the microbial community in Yeongam B changed between cultivation and harvest. In the three fields, Actinobacteria, Proteobacteria, Acidobacteria, Gemmatimonadetes, and Chloroflexi accounted for more than 80% of the bacteria, and they accounted for more than 93% in Yeongam A and B before harvest (Figure 4). Principal coordinate analysis (PCoA) based on Bray–Curtis dissimilarity revealed that the microbial communities before harvest were clearly clustered according to the cultivation regions (Figure 5). The plot showed that the microbial communities of Naju-2 differed from those of Yeongam A-2 and B-2, with the second PCoA axis corresponding to 15.64% of the variation.

### Virulence Test and Antioxidant Activity for Tubers Grown in the Three Fields

**Figure 6:**
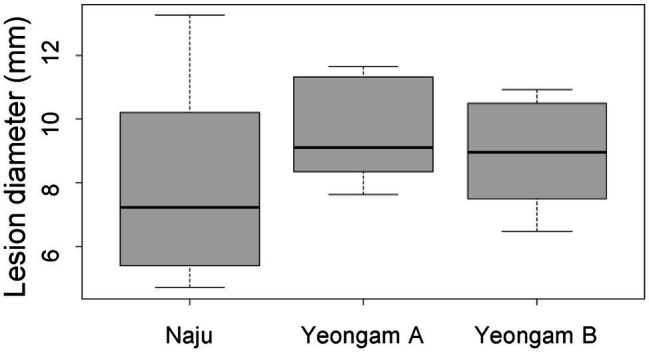
Lesion diameter by treatment with 10^6^ conidia/mL *F. solani* on sweetpotatoes grown in the three fields—Naju, Yeongam A, and Yeongam B. The box graph represents the interquartile range. The line across the box depicts the median (*n* ≥ 7).

To determine whether cultivation soil affects the defense mechanisms of sweetpotato tubers to root rot, the virulence test and antioxidant activity of sweetpotatoes grown in the three fields were investigated. A virulence test was used to measure the lesion size for the 10^6^ conidia/ml *F. solani* treatment in sweetpotato tubers (Figure 6). There was no significant difference in the lesion sizes of the sweetpotatoes tubers harvested from the three fields.

Before comparing the antioxidant activity against root rot of sweetpotatoes grown in three fields, we confirmed the antioxidant activity of root rot in sweetpotatoes (Supplementary Figure 5). TPC content, DPPH scavenging activity, and ABTS scavenging activity were different in the control and infected parts. TPC was highest in the infected part (2344.7 mg/100 g CGA) and lowest in the control part (402.5 mg/100 g CGA). ABTS scavenging activity was the highest in the infected part (96.8%) and the lowest in the control part (29.7%). The middle part had values between those of the other two parts. There was no significant difference in DPPH scavenging activity between the infected and middle parts. However, it showed the lowest value in the control part.

**Figure 7:**
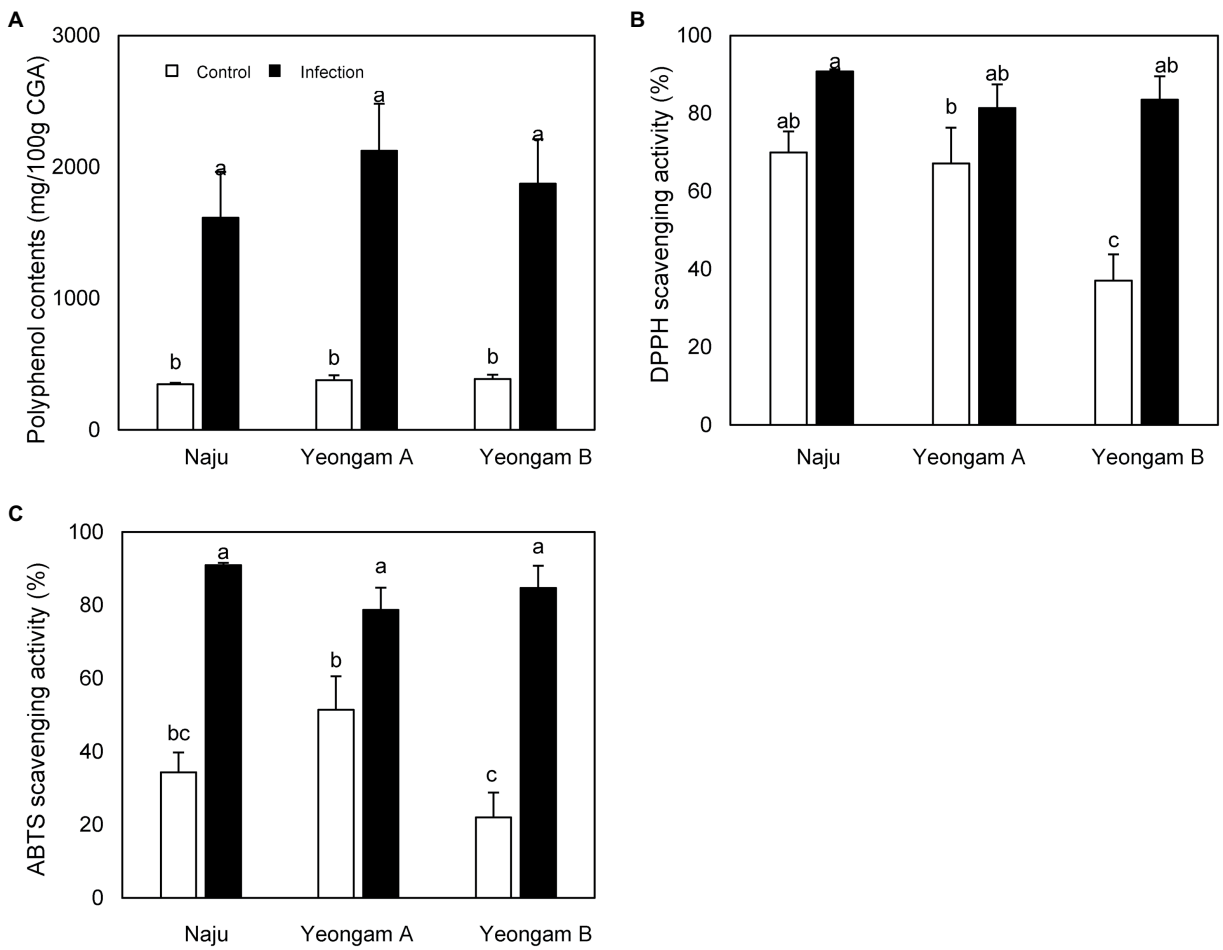
Antioxidant activity of sweetpotatoes infected with root rot in the three fields—Naju, Yeongam A, and Yeongam B. **(A)** Total polyphenol contents. **(B)** DPPH scavenging activity (%). **(C)** ABTS scavenging activity (%). Data are expressed as mean ± S.D. (*n* ≥ 4). Each value of different letters (a–c) above bars is significantly different by Duncan’s multiple range test at *p* < 0.05.

TPC content, DPPH scavenging activity, and ABTS scavenging activity of sweetpotatoes infected with root rot harvested from the three regions were also higher than those of uninfected sweetpotatoes (Figure 7). However, there were no significant differences between the infected sweetpotatoes. In contrast, uninfected sweetpotatoes demonstrated significant differences in DPPH and ABTS scavenging activity across the three fields. In particular, Yeongam B had the lowest values of scavenging activity in uninfected sweetpotatoes.

## Discussion

### Soil-Derived Infection of Root Rot in Sweetpotato

In many plants, root rot is induced by the soil-borne pathogen *Fusarium* spp. (Ozbay and Newman, 2004; Beccari et al., 2011; Chang et al., 2015). Likewise, it has been presumed that the root rot in sweetpotatoes is induced by cultivating soils because of *Fusarium* spp. isolated from the root rot (Scruggs and Quesada-Ocampo, 2016; Yang et al., 2018). However, as the extent of root rot of sweetpotatoes is more during storage periods after harvest than during cultivation periods, it had not been clarified whether *Fusarium* spp. in the cultivation soil actually induces root rot in harvested sweetpotatoes.

The difference in the incidences of root rot between the three fields in this study (Figure 2) suggests the possibility of root rot in sweetpotato by cultivating soil. This was because fields were managed under the same curing and storage conditions and experienced similar weather conditions. Although Supplementary Figure 3 illustrates no significant differences among the fields, the abundances of *Fusarium* spp. in the fields were different. Therefore, root rot of sweetpotato might be caused by *Fusarium* spp. in the cultivating soil or by the characteristics of the cultivating soil. In addition, some studies have suggested that the incidence of root rot caused by *Fusarium* spp. is affected by the soil physicochemical properties. Root rot induced by *F. graminearum* in soybean is more common when cultivated in sand than in loam and is known to occur more in soils with a pH of 6 than a pH of 8 (Cruz et al., 2019). Duffy and Défago (1999) indicated that the absorbed form of nitrogen increased the severity of tomato root rot. Furthermore, Triky-Dotan et al. (2005) showed that irrigation with saline water resulted in a higher severity of tomato root rot.

Sweetpotatoes treated with *Fusarium*-cultivated soils showed symptoms of root rot during storage periods (Figure 1). This result indicated that *Fusarium* spp. in the cultivating soil infected sweetpotatoes stored after harvest. However, accurately estimating the time of intrusion of *Fusarium* spp. is difficult. *Fusarium* spp. have been known to invade through wounds received during the harvesting process; therefore, infection may occur from residual soil between the harvesting and curing treatment (Clark, 1980). However, the storage temperature (13–15°C) of sweetpotatoes is not suitable for the growth conditions (25–30°C) of *Fusarium* spp. Therefore, root rot caused by *Fusarium* spp. in residual soil can be slow to occur during long storage periods (Scruggs and Quesada-Ocampo, 2016; Cruz et al., 2019). Further studies are required to clarify the time of infection by *Fusarium* spp. and the development of root rot between harvesting and curing treatments.

### Involvement of the Microbial Community in the Incidence of Root Rot

Among three investigated fields, the richness and diversity of the microbial community differed according to the region and cultivation period (Figure 3). In particular, Yeongam B, which had a high incidence of root rot, had a decrease in the diversity of the microbial community according to the cultivation period. Before harvest, the diversity was lower than that of Naju, which had a low incidence of root rot. This result was consistent with the finding of a lower OTU abundance and Shannon index of bacterial communities compared to those of healthy samples in root rot-infected tobacco plants (Zheng et al., 2021). Some studies have indicated that microbial diversity influences soil quality, pathogen defense mechanisms, and the promotion of plant growth by increasing nutrient uptake (van Elsas et al., 2002; Jaiswal et al., 2017). Therefore, it is suggested that various microorganisms in the cultivation soil have a significant influence on the yield and growth of sweetpotato tubers.

Actinobacteria, Proteobacteria, Acidobacteria, Gemmatimonadetes, and Chloroflexi accounted for more than 80% of the total bacteria among the three fields, and for more than 93% found in Yeongam A and B before harvest (Figure 4). This result indicated an increase in the ratio of dominant bacteria species by the cultivation of sweetpotatoes in the Yeongam regions, where the incidence of root rot was high, which may have also contributed to the lower diversity of the microbial community in Yeongam B as shown in Figure 3. In Naju, where the incidence of root rot was low, Actinobacteria, Acidobacteria, Gemmatimonadetes, and Chloroflexi increased, and Proteobacteria were low before harvest. In contrast, Yeongam B showed the opposite pattern. This result was consistent with increases in the amount of Acidobacteria and Actinobacteria and decreases in that of Proteobacteria in healthy Panax notoginseng plants compared to those of diseased plants with root rot disease (Zhang et al., 2020). Klein et al. (2013) also showed that in experiments with cucumber roots, the amount of Actinobacteria was high and that of Proteobacteria was low in suppressive soils compared to those in control soils. These results agree with the findings of many studies demonstrating the potential of Actinobacteria as a biocontrol (Misk and Franco, 2011; Palaniyandi et al., 2013).

### Defense Response to Root Rot of Sweetpotatoes Grown in Three Study Regions

Plant diseases are caused by the pathogenicity of the fungus and the resistance of the host plant (Flor, 1971). As described above, in the three fields with different incidence rates, the distribution of *Fusarium* spp. and microbial communities differed according to the cultivation region and cultivation period (Supplementary Figure 3; Figure 5). However, the defense response of sweetpotatoes to root rot according to the cultivating soil has not been elucidated.

Few studies have been conducted on the defense mechanisms of root rot in sweetpotato. In sweetpotatoes, the polyphenol content and antioxidant activity increased during infection and was defense response to invading *Fusarium* spp. (Supplementary Figure 5). This defense response was also confirmed in studies on the biotic stress of sweetpotatoes. For example, an increase in phenolic content and antioxidant capacity has been reported following infection with *C. fimbriata* and invasion of weevils in sweetpotatoes (Liao et al., 2020; Mohsin et al., 2021).

When sweetpotato tubers harvested from three study fields were treated with the same concentration (10^6^ conidia/ml) of *F. solani*, there was no significant difference in lesion diameter (Figure 6). In addition, tubers with root rot showed no significant differences in polyphenol content and antioxidant activities across the three fields (Figure 7). These results imply that the difference in the incidence rates of root rot was either (1) due to a variation in the concentration of *Fusarium* spp. or (2) caused by defense mechanisms other than the antioxidant capacity and polyphenols functions in the three regions. Some studies have shown differences in the resistance of plants by beneficial microorganisms according to soil characteristics. Imperiali et al. (2017) reported that soil texture and nutrients affected the expression of antimicrobial genes in representative Swiss agricultural soils. In addition, the initial soil microbial community affects the plant–pathogen interactions and thus determines the growth of tomatoes (Wei et al., 2019). Siegel-Hertz et al. (2018) showed different soil microbial communities when comparing *Fusarium* wilt-suppressive soil and conducive soil in flax cultivation areas. In this study, artificially induced root rot presented a lesion diameter that was 2 weeks after treatment with *F. solani*, the effect on long-term resistance is unknown. Furthermore, defense mechanisms induced by other mechanisms, apart from that by the antioxidant activity and polyphenol functions, should be investigated in future studies.

## Conclusion

Root rot, a storage disease fatal to sweetpotato yield, is induced by *Fusarium* spp., a soil-borne pathogen. However, studies that prove root rot by pathogens in cultivating soil and characteristics of cultivating soil are insufficient. In this study, root rot in sweetpotatoes treated with soil containing *F. solani* and *F. oxysporum* before the storage period was investigated, and the difference in the incidences of root rot of sweetpotato among three fields with similar storage conditions was confirmed. These results suggest that the characteristics of the cultivation soil are important for the incidence of root rot of sweetpotato. In addition, the involvement of soil microorganisms in sweetpotato cultivation and the incidence of root rot disease according to the cultivation period were confirmed. Therefore, considering cultivating soil and soil microorganisms is necessary to improve sweetpotato yield through the control of root rot of sweetpotato.

## Data Availability Statement

The datasets presented in this study can be found in online repositories. The names of the repository/repositories and accession number(s) can be found at: https://www.ncbi.nlm.nih.gov/, PRJNA807739.

## Author Contributions

SK designed the experiments and wrote the manuscript. SK and TK collected soil samples and performed virulence tests in the three study fields. All authors contributed to the article and approved the submitted version.

## Funding

This study was supported by the Basic Research Program (Project No. PJ01605801) funded by the Rural Development Administration of the Republic of Korea.

## Conflict of Interest

The authors declare that the research was conducted in the absence of any commercial or financial relationships that could be construed as potential conflicts of interest.

## Publisher’s Note

All claims expressed in this article are solely those of the authors and do not necessarily represent those of their affiliated organizations, or those of the publisher, the editors and the reviewers. Any product that may be evaluated in this article, or claim that may be made by its manufacturer, is not guaranteed or endorsed by the publisher.
