# Supplementary material for: Incidence Rates of Root Rot in Sweetpotato Caused by Cultivation Soil and Soil Microorganisms During Storage Periods
Source: Front Plant Sci. 2022 May 3;13:897590. doi: 10.3389/fpls.2022.897590 (PMC9113054; doi:10.3389/fpls.2022.897590)
Supplement: Supplementary file 1 [file Data_Sheet_1.PDF]

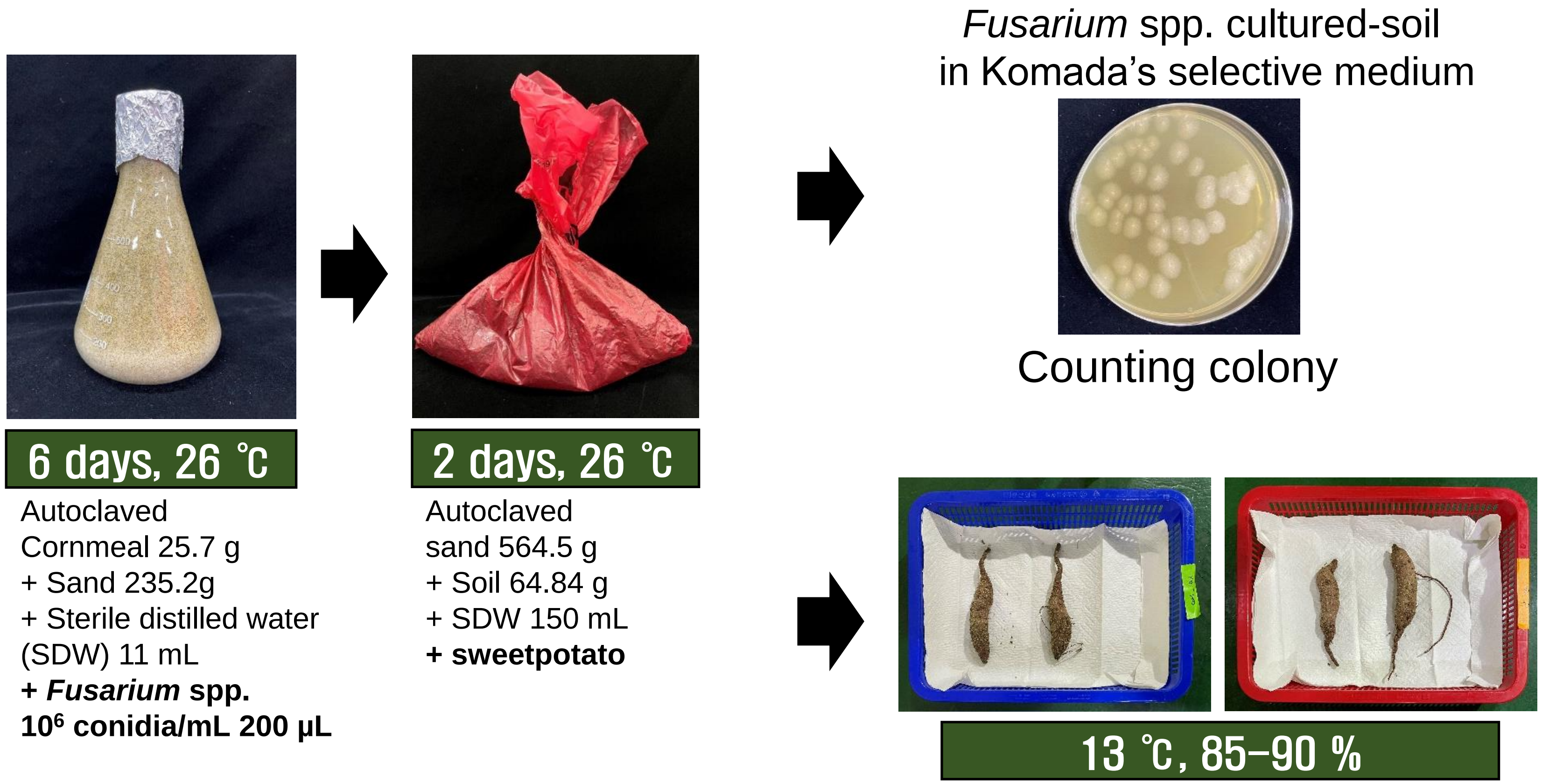

Supplementary Figure 1. A schematic diagram of soil inoculation. Soil containing *Fusarium* spp. caused root rot of sweet potatoes. The pathogen concentration in the soil was measured by Komada's selective medium.

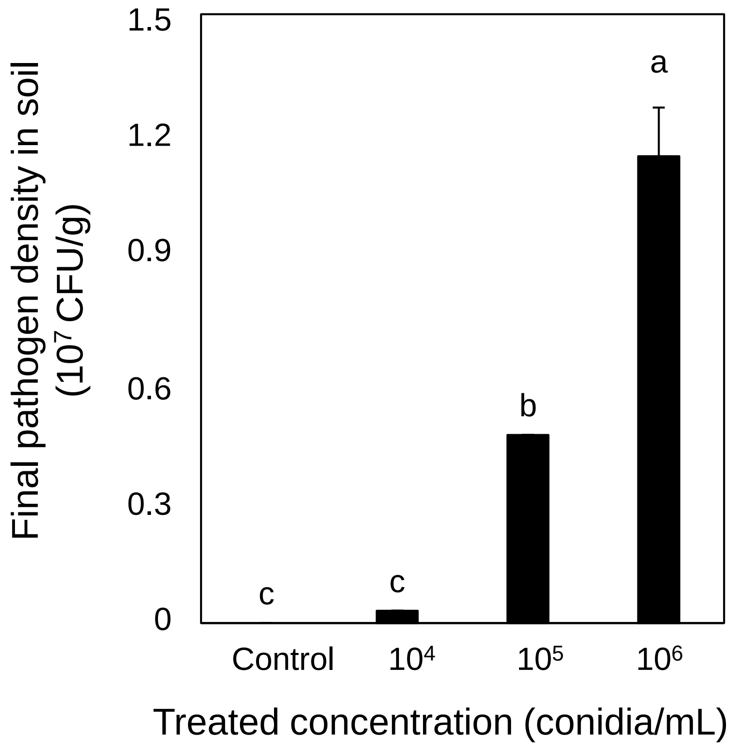

Supplementary Figure 2. *Fusarium solani* from soil inoculation. Abundance of *F. solani* treated concentration shows the final concentration of *F. solani* cultured in the soil. Final abundance indicates the number of colonies by treated soil on Komada's selective medium. Data are expressed as mean ± S.E. (n≥2). Each value of different letters (a–c) above bars is significantly different by Duncan's multiple range test at $p < 0.05$.

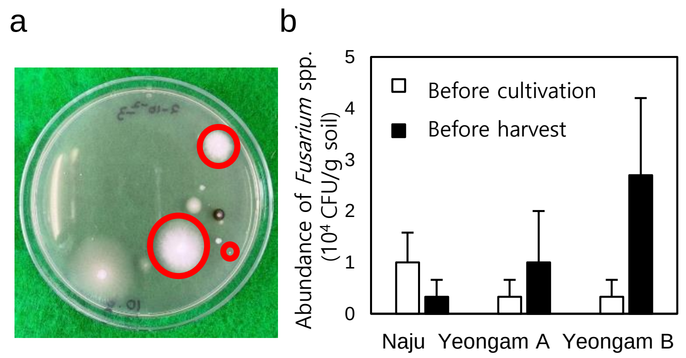

Supplementary Figure 3. *Fusarium* spp. in three fields – Naju, Yeongam A, and Yeongam B. (A) *Fusarium* spp. cultured from cultivated soil on the Komada's selective medium. (B) Abundance of *Fusarium* spp. in three fields. Red circles indicate colonies identified as *Fusarium* spp. by the ITS sequence. Data are expressed as mean ± S.E. (n=3).

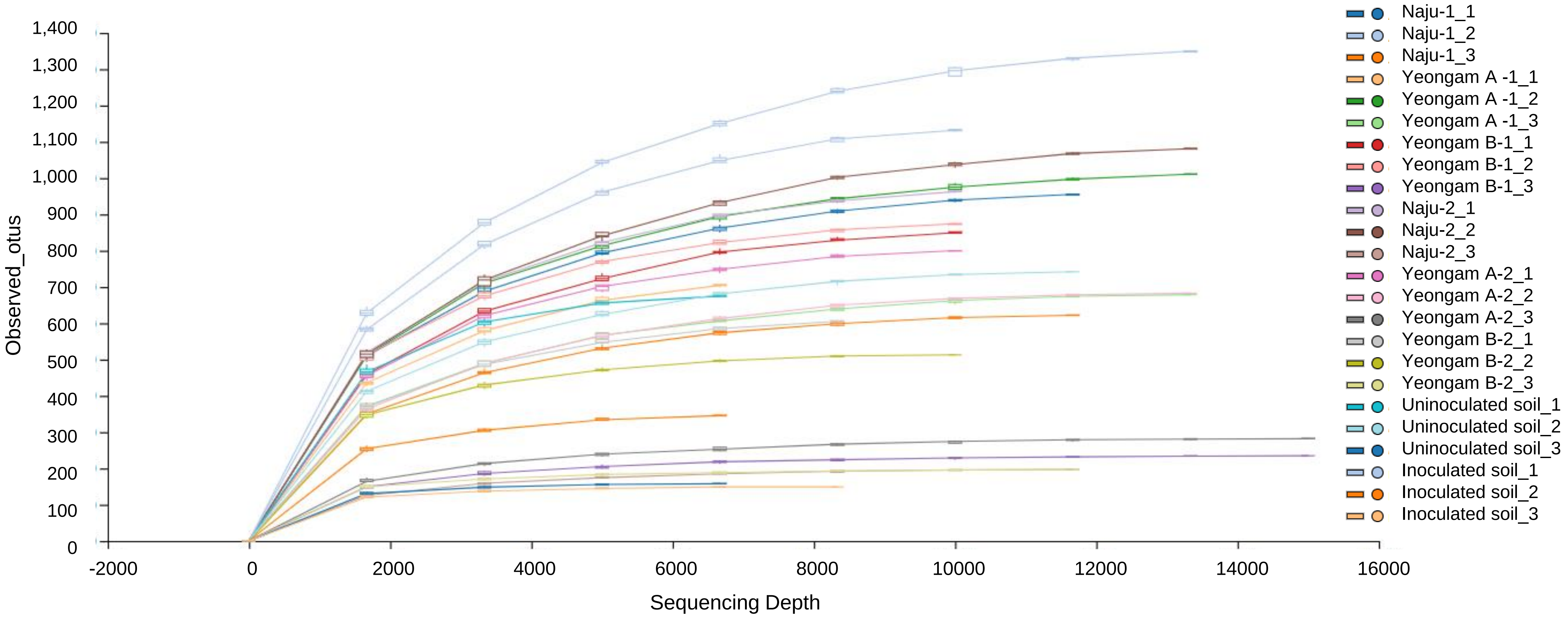

Supplementary Figure 4. Rarefaction curves for soil samples. 1-2 indicate before planting and harvest, respective. 1-3 represent three replicates samples performed in this study.

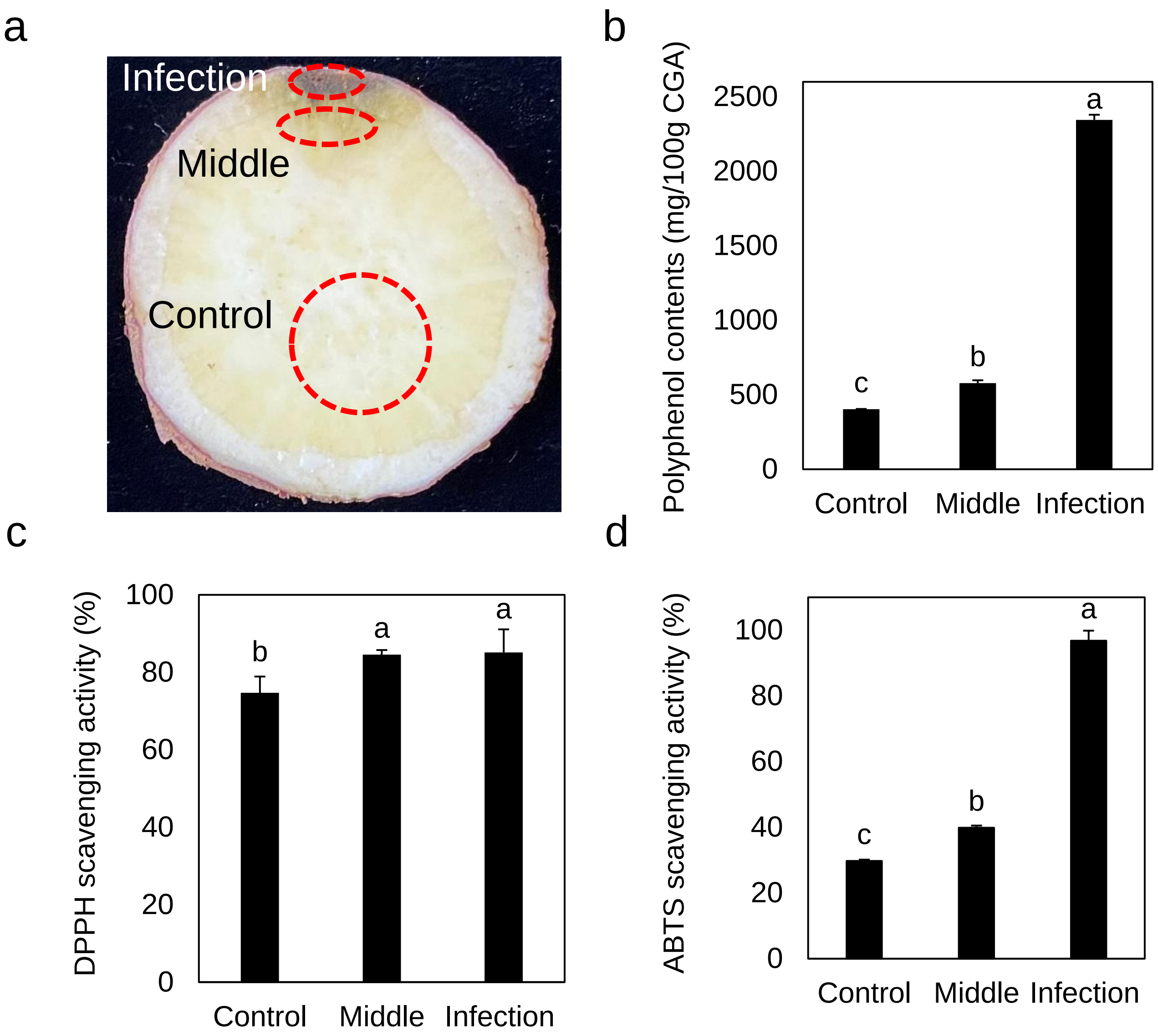

Supplementary Figure 5. Antioxidant activity by root rot in sweetpotato. (A) Sample parts in the cross-section of sweetpotato. (B) Total polyphenol contents. (C) DPPH scavenging activity (%). (D) ABTS scavenging activity (%). CGA indicates chlorogenic acid. Data are expressed as mean ± S.D. (n = 3). Each value of different letters (a–c) above bars is significantly different by Duncan's multiple range test at $p < 0.05$.
